# Perceived psychological stressors in female age-specific national team players

**DOI:** 10.3389/fpsyg.2026.1803701

**Published:** 2026-06-03

**Authors:** Stig Arve Sæther, Joanne Butt, Martin Eubank, Ingar Mehus, Rune Høigaard, Nils Petter Aspvik

**Affiliations:** 1 Department of Sociology and Political Science, Norwegian University of Science and Technology (NTNU), Trondheim, Norway; 2 Liverpool John Moores University, Liverpool, United Kingdom; 3 Department of Sport Science and Physical Education, University of Agder, Kristiansand, Norway

**Keywords:** age, evaluative concerns, personal strivings, playing time, stressors

## Abstract

**Introduction:**

Psychological stressors have become a natural part of the process of developing skills in talent development environments of youth athletes. However, there is little research on psychological stressors among talented female athletes and how this is impacted by an athletes’ perfectionism. This study investigated how female national team players in football, handball, and ice-hockey in Norway (controlled for age, sport, overload injury and playing time) experienced perceived psychological stressors (PPS) and how the two perfectionism dimensions - evaluative concerns (EC) and personal strivings (PS) impact on PPS.

**Methods:**

The sample in this study was 145 female (Handball 27%, Ice-Hockey 43% and Football 30%) players selected to an age-specific national team of U16-U19 (*M* = 16.01, SD = 1.19). Cronbach’s alpha was calculated to test the scale reliability of the factors; PPS (*α* = 0.88), EC (*α* = 0.84), PS (*α* = 0.78). A linear regression analysis was conducted with PPS as the dependent variable.

**Results:**

The linear regression analysis with PPS as the dependent variable, with the independent variables EC, PS, self-reported playing time, OI and age, explained 25% of the variance in PPS and was statistically significant (*p* < 0.001). EC (*p* < 0.01) and OI (*p* < 0.05) had a significant positive association with PPS. Handball players scored significantly lower on PPS compared with ice-hockey players (*p* < 0.01) and football players (*p* < 0.05). There was however no significant difference in PS, age, and playing time on PPS.

**Discussion:**

The current study highlights the importance of key stakeholders being aware that athletes who score high on EC are more vulnerable to everyday stress, and that athletes are particularly vulnerable to stress when they are injured.

## Introduction

Talent development has been found to be complex, with many factors and actors, including psychological characteristics, impacting on the talent development process (Höner et al., 2023). Two of the psychological characteristics impacting on the athletes´ development are psychological stressors and perfectionism. Experiencing psychological stressors is a natural part of the process of developing skills in talent development environments of youth athletes (Olsson et al., 2021). According to the transactional and interactional stress perspective, stressors occur during individuals’ transactions with their environments and have been defined as “environmental demands (i.e., stimuli) encountered by an individual” (Lazarus, 2000, p. 329). Throughout the talent development process, young talented athletes will encounter a variety of challenges (e.g., less playing time, injuries) and demands (perceived expectations) both from themself and from key actors within (e.g., coaches, teammates) and outside (e.g., school, parents) their sporting environment. Consequently, psychological stressors have been found to be a natural part of the athletes´ talent development process (Rye et al., 2022), even though male and female athletes experience these stressors differently (Rye et al., 2022; Selbekk et al., 2025). Female athletes have, for instance, been found to experience more interpersonal events as stressors compared to males (Rudolph, 2002; Rudolph and Hammen, 1999). The number of studies on female athletes is however scarce, as the research on talent development has mainly focused on male athletes (Curran et al., 2019; Hauser et al., 2024), which is aligned with the notion of a gender gap in sport research (Cowley et al., 2021). Still, a newly published study (Selbekk et al., 2025) on female junior and senior players revealed that the players experienced several sport stressors, such as disparities in performance levels between the junior and senior teams, especially during matches, and high expectations and demands from coaches. Non-sport stressors included struggles to balance football with social life and education, especially where education was their backup plan. Several studies have also reported that male players with limited playing time experienced significantly higher levels of stress compared to those who experience a high degree of playing time (Sæther and Aspvik, 2016; Engan and Sæther, 2018).

According to Curran and Hill (2019), there is a growing societal trend for adolescents to be harder on themselves and report more social pressure and expectations than previous generations. Nevertheless, perfectionism has sometimes been seen as crucial to achieving success (Hardy et al., 2017). Thus, perfectionism appears to be inherent in young ambitious athletes, particularly among those who have attained a high-performance level at an early age, such as national team athletes (Klund and Sæther, 2017). Perfectionism is often characterized by unrealistically high expectations and overly critical self-evaluations. Perfectionistic concerns are associated with a multitude of negative mental health outcomes, including psychological stressors (and other negative mental health outcomes, such as depression, anxiety disorders, obsessive-compulsive disorder, and eating disorders) (Egan et al., 2011). Perfectionism can be dichotomized into *perfectionistic strivings* (PS) (e.g., an internal pressure to strive for perfectionism) and “*evaluative concerns*” (EC) (e.g., being overly concerned with the implications of imperfection) (Frost and Marten, 1990). PS more often correlates with positive mental health outcomes (Stoeber and Otto, 2006), and EC is often referred to as maladaptive or unhealthy perfectionism (Hill et al., 2018).

While the research has shown that stress and perfectionism separately impact athletes talent development process (Kim et al., 2025), there is little research on psychological stressors among talented athletes and how this is impacted by an athletes’ perfectionism (McLoughlin et al., 2022). One of few studies on the relationship between perfectionism and stress on athletes indicate that athletes high in perfectionistic concerns are likely to experience heightened levels of stress in sport (Olsson et al., 2021). Based on the research gap exploring the relationship between perfectionism and stress (McLoughlin et al., 2022) and aligned with the general gender gap in sport research (Cowley et al., 2021), the main aim of this study was to investigate female age-specific national team players perception of stressors and its relationship with perfectionism. Earlier research has found between-sport differences in the perceived talent development environment (Lyons et al., 2024; Mehus et al., 2025), while no study has looked at such between-sport differences related to psychological stressors, indicating the novelty of the present study. Lastly, since lack of playing time and injuries have been found to impact athletes’ perceptions of stressors (Giske et al., 2021; Torvik et al., 2025), the study aimed to investigate whether playing time and overload injury (OI) impact on perceived psychological stressors (PPS). More specifically, the present study established the following hypothesis: (a) EC are expected to have a significant positive association with PPS, and PS a negative association with PPS, (b) less playing time is related to more PPS, and (c) OI are expected to have a significant positive association with PPS. Furthermore, this study will exploratively examine potential variations in perceived stress across the three sports from which participants are drawn. By comparing diverse sporting environments, we aim to identify whether specific athletic contexts contribute uniquely to the stress experiences of athletes beyond the primary variables of interest.

## Methods

To examine female age-specific national team players experience of PPS and how this is impacted by perfectionism, we recruited players from football, handball and ice hockey.

### Participants, recruitment, and procedure

The sample in this study consisted of all athletes who were recruited to participate in a national team training camp in their respective sports. Subsequently 145 female (Handball 28%, Ice-Hockey 41% and Football 31%) players (*M* = 16.01, SD = 1.19) were recruited to the study. Data was collected during a one-week training camp for the respective sports and conducted during the players´ lunch break to minimize the interference on each team’s training plan. The questionnaire took approximately 15 min to complete and was administered by hand by one of the researchers in the research group. The players were also given instructions about the questionnaire itself (e.g., the different sections), before completion, and after they received information about the aim of the study and their rights as participants.

### Instruments and measures

#### Stress

To measure perceived psychological stress the Athlete Stressors Questionnaire (ASQ) was used (Sæther, 2017), measuring a global stressor score across 12 items. Two examples of an item were: “How stressful is it to be evaluated by your coach” and “How stressful is it to keep up in school”. Players were asked to respond to a Likert-designed scale from 1–5; 1 (*not stressful at all*); 2 (*a little stressful*); 3 (*moderately stressful*); 4 *(stressful*); and 5 (*very stressful*). The Cronbach’s *α* coefficient shows good internal consistency (α = 0.88) (Cortina, 1993).

#### Perfectionism

The Multidimensional Perfectionism Scale – Brief (FMPS–brief) (Burgess et al., 2016) consists of a total of eight questions, with two subscales: Evaluative concerns (EC) *(4 items,* i.e.*, the fewer mistakes I make, the more people will like me)* and personal strivings (PS) *(4 items,* i.e.*, I have extremely high goals)*. The items are scored on a Likert scale ranging from 1 (*strongly disagree*) to 5 (*strongly agree*). The Cronbach’s *α* coefficient shows good internal consistency on both EC (α = 0.84), and PS (α = 0.78).

#### Self-reported playing time

To assess self-reported playing time the participants answered the following question: “How much playing time did you have in your club last season, using the following categories: 1 (*all matches*), 2 (*most matches*), 3 (*some of the matches*) and 4 (*few matches*). The distribution of self-reported playing time was 1 = 61%, 2 = 25%, 3 = 7%, and 4 = 7%. The variable was dichotomized into 0 (*not all matches*) and 1 (*all matches*) before analysis.

#### Overload injury

Overload injury (OI) was measured using the following question: Are you struggling with sports-related overload injury now, with responses on a three-point scale, ranging from 1 (*no*), 2 (*yes, in some degree*), and 3 (*yes, to a high degree*). The OI distribution in the current study was 1 = 63.5%, 2 = 33% and 3 = 3.5%. Based on the skewed distribution we merged category 2 and 3. The variable was dichotomized into 0 (*no OI*) and 1 (*OI*) before analysis.

### Data analysis

Responses from the questionnaires were entered manually into Excel before being imported into STATA/SE 19.5.0 (StataCorp, TX). A series of two-way ANOVAs were conducted to examine the effects of sport type on psychological stress, perfectionism, playing time, and injury status (overload injury) (Tables 1, 2). Preliminary assumptions were tested using Levene’s test for homogeneity of variances and the Shapiro–Wilk test for normality. Although minor deviations from normality were observed for certain variables, visual inspection of Q–Q plots and the recognized robustness of ANOVA for moderate sample sizes (Schmider et al., 2010) justified its continued use. Effect sizes were reported as partial eta squared, which facilitates within-study comparisons (Fritz et al., 2012). These values were interpreted using Cohen’s (1977) benchmarks: small (0.01), medium (0.06), and large (0.14). *Post hoc* comparisons using the Tukey’s HSD test were also calculated, allowing the comparison of between sport differences. Pearson’s chi-square tests were used to assess the associations between categorical variables (Table 2). Due to the non-normal distribution of certain variables, Spearman’s rho was used to assess the monotonic associations between all variables (Table 3) (Field, 2018). Cramér’s V was employed to assess the strength of the association between the categorical variables, following the initial chi-square analysis.

**Table 1:** Psychological stressors, across sports.

Psychological stressors^a^	Handball (*n* = 40) M (SD)	Ice-hockey (*n* = 60) M (SD)	Football *n* = 45) M (SD)	Total (*n* = 145) M (SD)	Eta-squared	Alpha deleted
Pressure regarding schoolwork	3.70 (1.30)	3.74 (1.28)	4.04 (1.13)	3.82 (1.24)	0.01	0.87
To keep up in school	3.68 (1.31)	3.62 (1.30)^d^	4.18 (0.96)^c^	3.80 (1.23)	0.04*	0.87
Match performance	3.60 (1.35)	3.68 (1.08)	3.51 (1.21)	3.61 (1.19)	0.00	0.86
Coaches’ high expectations	2.75 (1.37)^c, d^	3.82 (0.99)^b^	3.42 (0.97)^b^	3.41 (1.18)	0.14**	0.87
Being evaluated by your coach	2.80 (1.29)^c^	3.60 (1.20)^b^	3.07 (1.07)	3.22 (1.23)	0.08*	0.85
Pressure yourself to achieve goals	3.18 (1.22)	3.27 (1.29)	3.04 (0.93)	3.18 (1.16)	0.01	0.86
Not enough time to invest in football	2.78 (1.42)	3.35 (1.35)	3.24 (1.30)	3.16 (1.37)	0.03	0.87
Training performance	2.68 (1.33)	3.17 (1.13)	3.04 (1.07)	3.00 (1.18)	0.03	0.86
Being evaluated by teammates	2.40 (1.34) ^c^	3.51 (1.12)^b, d^	2.71 (1.27)^c^	2.96 (1.31)	0.13**	0.86
Team selection	2.15 (1.33)^c, d^	3.27 (1.49)^b^	3.13 (1.12)^b^	2.93 (1.42)	0.11**	0.86
Disagreement with your coach	2.18 (1.43)	2.43 (1.31)	2.69 (1.36)	2.44 (1.37)	0.02	0.87
To keep up in training	1.85 (0.98)^c^	2.62 (1.24)^b^	2.24 (1.13)	2.30 (1.17)	0.07**	0.86
Average (Global stress)	2.81 (0.96)^c^	3.34 (0.76)^b^	3.19 (0.67)	3.15 (0.82)	0.07*	0.88

Sorted from highest to lowest (*n* = 145).

*p < 0.05; **p < 0.01, Statistical difference between sports.

^a^Psychological stressors scale: 1 = not stressful, 2.5 = moderately stressful, 5 = very stressful, ^b^ Statistically significant different from Handball (One-way Anova, *p* < 0.05), ^c^Statistically significant different from Ice-hockey (One-way Anova, *p* < 0.05), ^d^Statistically significant different from Football (One-way Anova, *p* < 0.05).

**Table 2:** Age, perfectionism, playing time and injury situation, across sports (*n* = 145).

	Handball (*n* = 40)	Ice-hockey (*n* = 60)	Football (*n* = 45)	Total (*n* = 145)	Eta-squared
	M (SD)	M (SD)	M (SD)	M (SD)	
Age	16.43 (0.81)^c^	15.27 (1.00)^b, d^	16.71 (1.12)^c^	16.01 (1.19)	0.31**
Perfectionism^a^					
Evaluative concerns	2.91 (1.04)	2.66 (1.17)	2.85 (0.97)	2.79 (1.07)	0.01
Personal strivings	4.02 (0.82)^c^	3.48 (0.87)^b, d^	4.14 (0.72)^c^	3.83 (0.86)	0.12**

	Percentage	Percentage	Percentage	Percentage	Cramér’s V
Playing time (all matches)	75.00%	67.19%	44.44%	62.42%	0.25**
Overload injury (yes now)	52.50%	28.33%	33.33%	36.55%	0.21*

*p < 0.05 **p < 0.01, Statistical difference between sports.

^a^Psychological stressors scale: 1 = not stressful, 2.5 = moderately stressful, 5 = very stressful, ^b^Statistically significant different from Handball (One-way Anova, *p* < 0.05), ^c^Statistically significant different from Ice-hockey (One-way Anova, *p* < 0.05), ^d^Statistically significant different from Football (One-way Anova, *p* < 0.05).

**Table 3:** Spearman correlation analysis of global stress and independent variables (*n* = 145).

	1	2	3	4	5	6	7	8	9
1. Global stress^a^	–								
2. Age	−0.06	–							
3. Handball (yes)	−0.21**	0.22**	–						
4. Ice-hockey (yes)	0.15*	−0.54**	−0.52**	–					
5. Football (yes)	0.04	0.36**	−0.41**	−0.56**	–				
6. Evaluative concerns^b^	0.35**	0.05	0.06	−0.09	0.04	–			
7. Personal strivings^b^	0.08	0.02	0.13	−0.35*	0.24**	0.32**	–		
8. Playing time (All matches)	−0.12	−0.28**	0.17*	0.06	−0.23**	−0.06	0.09	–	
9. Overload injury (Yes)	0.09	0.13	0.20**	−0.14	−0.05	−0.08	0.09	−0.05	–

*p < 0.05; **p < 0.01.

^a^Global stress scale: 1, Not stressful – 5, Very stressful. ^b^Perfectionism scale: 1, strongly disagree – 5, strongly agree.

Preliminary diagnostics were conducted to ensure the data met the assumptions of Ordinary Least Squares (OLS) regression analysis. Multicollinearity was assessed using Variance Inflation Factors (VIF), with all values falling well below the recommended threshold of 5, indicating no significant overlap between predictors. Potential influential outliers were identified using Cook’s Distance; however, a sensitivity analysis revealed that these observations did not significantly alter the model’s coefficients. To address potential violations of the assumption of homoscedasticity (equal variance of residuals), the model was estimated using Huber-White robust standard errors [vce(robust)]. This approach was chosen to ensure the reliability of the values and confidence intervals, as robust standard errors provide unbiased estimates even in the presence of heteroscedasticity (Field, 2018; Hayes and Cai, 2007). Consequently, a robust OLS analysis was conducted with PPS as the dependent variable, while the independent variables were EC, PS, self-reported playing time, OI and age. In the analysis we controlled for type of sport (handball, football and ice-hockey). A *p*-value of <0.05 was used to define statistical significance.

## Results

Overall, 80% of the young talented female athletes reported perceived stress as being moderately stressful (PPS = 2.5), and one out of 10 reported it to be very stressful (PPS 4<) (Table 1). Moreover, young talented girls scored higher on PS compared to EC. Approximately 45% of athletes report having a high score (4<) on PS, while 15% report a high score on EC (4<). Pressure regarding schoolwork (mean 3.82) and to keep up in school (mean 3.80) were the most stressful variables among the psychological stressors, followed by match performance (mean 3.61) and coaches´ high expectations (mean 3.41) (Table 1). Furthermore, there was a significant correlation between PPS and EC, with no significant correlation between PPS and PS (Table 3). The linear regression analysis examining perfectionism, overload injury, playing time and age explained 25.2% of the variance in PPS and was statistically significant (*p* < 0.001) (Table 4). Athletes scoring higher on EC reported greater PPS (*p* < 0.01), while there was no association between PS and PPS. Furthermore, athletes reporting having an OI reported greater PPS compared to those who did not (*p* < 0.05). An association between playing time and PPS was not found, nor between age and PPS.

**Table 4:** Linear Regression analysis of Global stress and independent variables (*n* = 145).

Global stress^a^	Coefficient	Std. err.	Beta	*p*-value	95% conf. interval	
Age (13–18)	−0.01	0.06	−0.01	0.86	−0.13	0.11
Sports (ref: Ice-hockey)						
Handball	−0.64	0.21	0.39	0.01**	−1.06	−0.23
Football	−0.24	0.17	0.23	0.15	−0.58	0.09
Perfectionism^b^						
Evaluative concerns	0.28	0.07	0.36	0.01**	0.14	0.41
Personal strivings	0.05	0.08	0.06	0.47	−0.09	0.20
Playing time (All matches)	−0.18	0.11	−0.11	0.10	−0.41	0.04
Overload injury (Yes)	0.32	0.13	0.19	0.01**	0.07	0.57
Constant	2.57	1.00				
*R*-squared: 0.252 (25.2%), *F*-test: *p* < 0.01						

*p < 0.05; **p < 0.01.

^a^Global stress scale: 1, Not stressful; 5, Very stressful. ^b^Perfectionism scale: 1, strongly disagree – 5, strongly agree.

## Discussion

The aim of the current study was to investigate how young talented female age-specific national team players in Norway experience PPS and how this is impacted by perfectionism (PS and EC), and playing time, OI and age. School work and being evaluated by the coach or teammates are perceived as the most stressful factors, aligning with earlier research (Sæther and Aspvik, 2016). The athletes in the current study scored, on average, 3.2 on the PPS scale (above the scale mean), indicating that most athletes are balancing the challenges (not too many stressors at the same time) of their everyday life, which may reflect the holistic talent development environment in the Norwegian sport context (Gangsø et al., 2021; Mehus et al., 2025). The Holistic Ecological Approach (HEA) protects talented athletes from stress by addressing the ‘whole person’ (Stambulova et al., 2021). By integrating athletic, academic, and private domains, these environments provide a coordinated support network of coaches and peers. This systemic alignment offers the psychosocial resources needed to balance high training loads and life transitions effectively. However, it is important to acknowledge that some athletes will experience their environment as very stressful (Table 1), which underpins the importance of investigating how the independent variables are associated with athletes´ psychological stress.

Our first hypothesis stating that EC are expected to have a significant positive association with PPS, and PS a negative association with PPS, was partly supported since we only found a positive statistically significant association between EC and PPS (Table 4). Even though perfectionism has been argued to be essential to obtain success in sport (i.e., Hardy et al., 2017), our findings indicate that female athletes who score high on EC – being sensitive and vulnerable to evaluation in addition to being high on critical self-evaluation (Frost and Marten, 1990) – perceive higher levels of stress (Olsson et al., 2021). These findings indicate that coaches need to be sensitive to athletes high on EC. Our study found no association between personal strivings and perceived stressors among young talented athletes. These results suggest that high personal standards do not inherently lead to psychological stressors in this specific population. Consequently, striving for excellence may function as a neutral or even adaptive trait within elite youth sports environments. A newly published study has also highlighted how a perfectionistic climate may impede youth athletes’ development and experiences (Dargue-Fox et al., 2026). There is a need for such types of studies among high performing youth athletes, such as national team athletes.

Our second hypothesis stating that limited playing time would be associated with higher PPS, was not supported. While we expected regular starters to report lower stress, playing time did not emerge as a significant stressor for this group. This is likely because the sample consists of national team players who already secure substantial playing time at the club level, rendering it a less urgent concern (Sæther, 2017). These results deviate from established literature on youth academies, where playing time is often a primary source of psychological pressure (Sæther and Aspvik, 2016; Engan and Sæther, 2018). This suggests that for elite female athletes at this level, other factors may be more influential in driving stress than playing time itself. Consequently, the high level of established competence among national team players likely acts as a buffer, reducing the perceived threat typically associated with squad selection and competition for minutes.

Our third hypothesis stating that OI are expected to have a significant positive association with PPS was supported (Table 4). Injured players experience a higher level of stress compared to non-injured players, parallelling earlier research showing that being injured is perceived as a stressful situation (Torvik et al., 2025). This relationship likely reflects the taxing and uncertain nature of being injured, where the inability to train and compete undermines the athlete’s sense of competence and identity (Torvik et al., 2025).

Finally, comparing PPS between the included sports, our exploratory analyses showed that handball players reported significantly lower global stress than ice hockey players, and specifically experienced less stress regarding coaches’ expectations and team selection than both other groups. In contrast, ice hockey players felt significantly more pressure from teammate evaluations compared to both handball and football players. These findings are in line with Mehus et al. (2025) who stated that handball environments may protect athletes from external pressure. To better understand how young talented athletes experience psychological stressors, future research should employ diverse methods and perspectives across a wider range of sports.

### Strengths and limitations

While, to the authors knowledge, the current study is the first to examine how perfectionism associates with psychological stressors among junior national team athletes in the Scandinavian sporting context, the study has potential methodological limitations that are important to consider, such as cross-sectional design, sample characteristics, measurement limitations, and generalizability of the results. Due to the cross-sectional design, it is not possible to establish causal relationships between the variables. While including all players selected to the age-specific national teams in all three sports is considered a strength, investigating age-specific female national team players may elicit small sample sizes because of the number of players selected to national team camps. The measurements used in the present study have not been psychometrically validated, even though the PPS scale in the current study has been used in similar contexts (Sæther and Aspvik, 2016; Sæther, 2018). The FMPS–brief scale has not been used in the sport context, while the Self-reported playing time, and OI measurements consists of a limited number of items, even though they have been used in an earlier study (Sæther and Aspvik, 2016). We recognize using single-item measurements and reducing ordinal responses to binary variables before analysis may involve loss of information and could affect the results (Allen et al., 2022). We would therefore recommend future studies to include a more nuanced self-reported measure of playing time (i.e., Giske et al., 2021) or an objective assessment of both OI and playing time. Finally, the generalizability of the results is limited to the Norwegian context, even though there are potential similarities between age-specific national team players in the three included sports.

## Conclusion

This study demonstrates that perceived psychological stressors among elite female youth athletes is primarily driven by evaluative concerns and overload injury rather than competitive factors such as playing time. While high personal standards appear to be adaptive or neutral, Evaluative Concerns (EC) and being currently injured significantly increase perceived psychological stressor levels. Furthermore, the findings reveal that stress profiles are sport-specific; handball players benefit from environments that seem to buffer against coach-related pressure, whereas ice hockey players are more vulnerable to peer evaluation. Ultimately, these results suggest that the holistic approach in the Norwegian sports context generally supports athlete well-being, but targeted interventions are needed for those with high self-criticism or those undergoing injury rehabilitation.

## Data Availability

The raw data supporting the conclusions of this article will be made available by the authors, without undue reservation.
